# Emergence of a Large-Plaque Variant in Mice Infected with Coxsackievirus B3

**DOI:** 10.1128/mBio.00119-16

**Published:** 2016-03-29

**Authors:** Yao Wang, Julie K. Pfeiffer

**Affiliations:** Department of Microbiology, University of Texas Southwestern Medical Center, Dallas, Texas, USA

## Abstract

Coxsackieviruses are enteric viruses that frequently infect humans. To examine coxsackievirus pathogenesis, we orally inoculated mice with the coxsackievirus B3 (CVB3) Nancy strain. Using HeLa cell plaque assays with agar overlays, we noticed that some fecal viruses generated plaques >100 times as large as inoculum viruses. These large-plaque variants emerged following viral replication in several different tissues. We identified a single amino acid change, N63Y, in the VP3 capsid protein that was sufficient to confer the large-plaque phenotype. Wild-type CVB3 and N63Y mutant CVB3 had similar plaque sizes when agarose was used in the overlay instead of agar. We determined that sulfated glycans in agar inhibited plaque formation by wild-type CVB3 but not by N63Y mutant CVB3. Furthermore, N63Y mutant CVB3 bound heparin, a sulfated glycan, less efficiently than wild-type CVB3 did. While N63Y mutant CVB3 had a growth defect in cultured cells and reduced attachment, it had enhanced replication and pathogenesis in mice. Infection with N63Y mutant CVB3 induced more severe hepatic damage than infection with wild-type CVB3, likely because N63Y mutant CVB3 disseminates more efficiently to the liver. Our data reinforce the idea that culture-adapted laboratory virus strains can have reduced fitness *in vivo*. N63Y mutant CVB3 may be useful as a platform to understand viral adaptation and pathogenesis in animal studies.

## IMPORTANCE

Coxsackieviruses frequently infect humans, and although many infections are mild or asymptomatic, there can be severe outcomes, including heart inflammation. Most studies with coxsackieviruses and other viruses use laboratory-adapted viral strains because of their efficient replication in cell culture. We used a cell culture-adapted strain of CVB3, Nancy, to examine viral replication and pathogenesis in orally inoculated mice. We found that mice shed viruses distinct from input viruses because they formed extremely large plaques in cell culture. We identified a single mutation, VP3 N63Y, that was sufficient for large-plaque formation. N63Y mutant viruses have reduced glycan binding and replication in cell culture; however, they have enhanced replication and virulence in mice. We are now using N63Y mutant CVB3 as an improved system for viral pathogenesis studies.

## INTRODUCTION

Coxsackievirus B3 (CVB3) is a nonenveloped RNA virus in the *Picornaviridae* family. CVB3 is implicated in a range of diseases, including myocarditis (1, 2), type 1 diabetes (2–4), and aseptic meningitis (5), causing >40,000 symptomatic infections each year in the United States (6). However, the majority of human CVB3 infections are mild or asymptomatic.

CVB3 capsids are formed by viral proteins VP1, VP2, VP3, and VP4, and binding to the coxsackievirus adenovirus receptor (CAR) is required for infection (7–9). In polarized epithelial cells, CVB3 infection requires an additional attachment receptor, decay-accelerating factor (DAF), because CAR is sequestered in tight junctions (10, 11). If CAR expression is low, as in some nonpolarized cell lines such as RD and Chinese hamster ovary (CHO) cells, CVB3 strains that do not bind DAF can evolve to use additional factors such as DAF and heparan sulfate (HS) to aid attachment efficiency (12–19).

RNA virus replication is error prone, giving rise to genetically diverse viral populations and facilitating the emergence of variants with altered properties, including cell attachment potential. An appropriate amount of viral population diversity is thought to aid viral adaptation to changing host environments and is required for full virulence *in vivo* (20–25). When viral populations encounter selective pressure, distinct viral variants can emerge. For example, many viruses used in laboratories today are cell culture-adapted variants. Cell culture adaptation reduces the virulence of many viruses and is frequently the basis of live-attenuated vaccine development. Some of these culture-adapted variants show changes in affinity for glycosaminoglycans (GAGs), which are sulfated polysaccharides, including HS and heparin. GAGs are present ubiquitously on cell surfaces (26). It has been shown that viruses from many different families interact with GAGs, which aids viral attachment. Viral affinity for GAGs is an important determinant of tissue tropism and pathogenicity (27–29).

To examine CVB3 replication and pathogenesis in mice, many studies use peripheral injection, which bypasses the normal oral route of infection, as a delivery method. CVB3 does not replicate efficiently in orally inoculated C57BL/6 mice, and orally inoculated immunocompetent C57BL/6 mice do not develop disease. While CVB3 replicates more efficiently in mice lacking the interferon alpha/beta receptor (IFNAR), the immune deficiency of these mice is not ideal for studies seeking to understand innate and adaptive responses to viral infection. Therefore, a better model system for oral CVB3 infections is needed since some effects, such as those imparted by intestinal microbiota, can only be uncovered by using the natural oral infection route (30–34).

In this study, we identified a large-plaque variant of CVB3 that emerged after oral inoculation of mice. We found that a single mutation, N63Y, in the VP3 protein was sufficient for the formation of large plaques. We characterized this variant both *in vitro* and *in vivo* and found that N63Y mutant CVB3 has a growth defect in tissue culture but is more pathogenic than wild-type (WT) CVB3 in mice. We also explored what factors promote the emergence of the large-plaque variant, the basis for large-plaque formation, and mechanisms underlying differences in viral replication and pathogenesis.

## RESULTS

### Emergence of a large-plaque variant of CVB3 following inoculation of mice.

**FIG 1:**
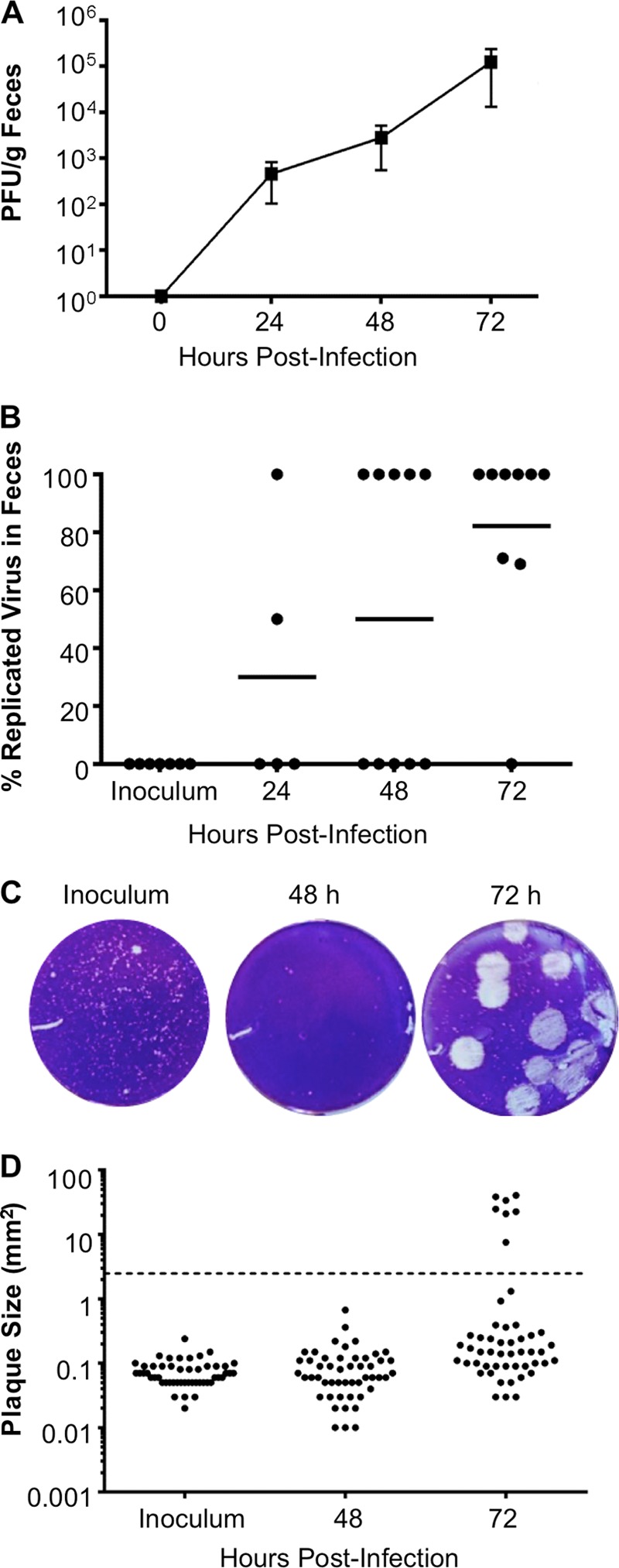
Emergence of a CVB3 large-plaque variant following oral inoculation. Four- to 6-week-old male IFNAR^−/−^ mice were orally inoculated with 5 × 10^7^ PFU of light-sensitive, neutral-red-labeled WT CVB3 in the dark, and fecal samples were collected at 24, 48, and 72 hpi in the dark. (A) Total viral titers determined by HeLa cell plaque assays with agar overlays. (B) Percentages of replicated (noninoculum) virus in fecal samples. Flowthrough inoculum viruses are light sensitive, whereas viruses that replicated in mice are light insensitive. Aliquots of each processed fecal sample were exposed to light or dark, and replication status was determined by dividing the number of PFU/ml of light-exposed samples by the number of PFU/ml of dark-exposed samples and multiplying by 100%. Each symbol represents one mouse. (C) Representative plaque morphologies of inoculum and 48- and 72-hpi fecal samples. (D) Plaque size quantification. Each symbol represents a plaque. Large plaques are defined as the average inoculum plaque size multiplied by a factor of 10 (2.473 mm^2^ or larger; indicated by a dashed line). For panels A and B, *n* = 5 to 10.

To examine the replication of CVB3 in the gastrointestinal tract, we orally inoculated immunodeficient C57BL/6 IFNAR knockout (IFNAR^−/−^ here) mice with 5 × 10^7^ PFU of neutral-red-labeled WT CVB3. Because mice shed unreplicated inoculum virus in their feces, it is difficult to quantify viral replication in the intestine in the first few days after oral inoculation. To avoid this problem, we examined viral replication by using light-sensitive, neutral-red-labeled virus. Virus propagated in the presence of neutral red dye is sensitive to light-induced inactivation by RNA cross-linking but loses light sensitivity upon replication in the dark inside mice, facilitating the assessment of replication and differentiating the inoculum virus from replicated virus in feces. Fecal samples were collected at 24, 48, and 72 h postinfection (hpi), and virus titers were determined by plaque assay using agar overlays (Fig. 1A). At 24 hpi, only two mice shed replicated virus while most of the mice shed replicated virus at 72 hpi (Fig. 1B). Surprisingly, plaques with morphology different from that of inoculum CVB3 were observed on 72-hpi fecal sample titer plates (Fig. 1C and D), suggesting that a variant emerged following viral replication in mice. Indeed, 0.67% of the fecal virus plaques were large at 48 hpi, while 11% were large at 72 hpi. We then examined the frequency of large-plaque viruses in our inoculum to determine whether the large-plaque variant was present prior to the inoculation of mice. We found that 0.26% of the inoculum CVB3 plaques had the large-plaque phenotype, suggesting that the large-plaque variant existed at a low level in the inoculum and was enriched following replication in the gut.

**FIG 2:**
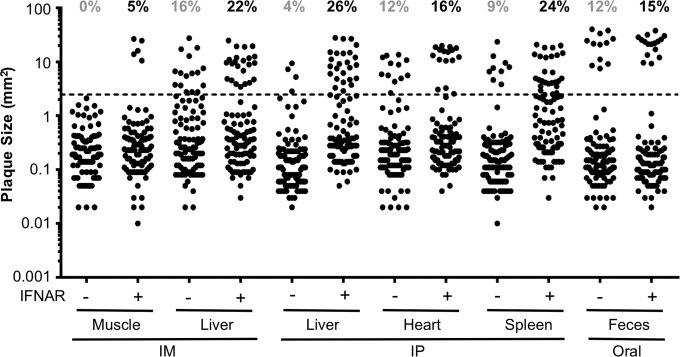
The emergence of the large-plaque variant differs in various tissues. Four- to 6-week-old male IFNAR^−/−^ or IFNAR^+/+^ mice were orally inoculated i.p. or i.m. with 5 × 10^7^ PFU of WT CVB3. Fecal samples were collected from orally inoculated mice at 72 hpi. Liver, heart, and spleen samples were harvested from i.p. inoculated mice at 48 hpi. Muscle and liver samples were harvested from i.m. inoculated mice at 48 hpi. Following processing, viruses were plated on HeLa cells by using agar overlays. The sizes of 100 randomly picked plaques were quantified with ImageJ. The total percentages of large plaques among hundreds of plaques per condition are indicated at the top. Large plaques are defined as the average inoculum plaque size multiplied by a factor of 10 (2.473 mm^2^ or larger; indicated by a dashed line).

To determine what factors contribute to the emergence of the large-plaque variant, we used different inoculation routes and examined the plaque morphology of virus from different tissues of immunocompetent or immunodeficient mice. IFNAR^−/−^ mice and immunocompetent IFNAR^+/+^ mice were orally, intraperitoneally (i.p.), or intramuscularly (i.m.) inoculated with 5 × 10^7^, 5 × 10^7^, or 2 × 10^6^ PFU of CVB3, respectively. Fecal samples were collected from orally inoculated mice at 72 hpi; livers, hearts, and spleens were harvested from i.p. inoculated mice at 48 hpi; and muscles and livers were harvested from i.m. inoculated mice at 48 hpi. We measured the virus plaque sizes and quantified the percentages of large plaques in different tissues (Fig. 2). First, we found that there were more large-plaque variants in IFNAR^+/+^ mice than in IFNAR^−/−^ mice. The increased emergence of the large-plaque virus in immunocompetent mice correlates with reduced viral replication, indicating that increased selective pressure may drive the emergence of the large-plaque variant. Second, large-plaque variants were present in feces from orally inoculated mice and visceral tissues from i.p. or i.m. inoculated mice but not in muscle from i.m. inoculated IFNAR^−/−^ mice. We hypothesize that increased selective pressure in the intestine and peritoneal cavity facilitated the emergence of large-plaque variants and/or the large-plaque variant has a fitness advantage in selected tissues. Overall, these results suggest that the large-plaque variant existed at a low level in our inoculum but was enriched following viral replication in several tissues of infected mice.

### A single amino acid change, VP3 N63Y, is sufficient to confer the large-plaque phenotype.

**FIG 3:**
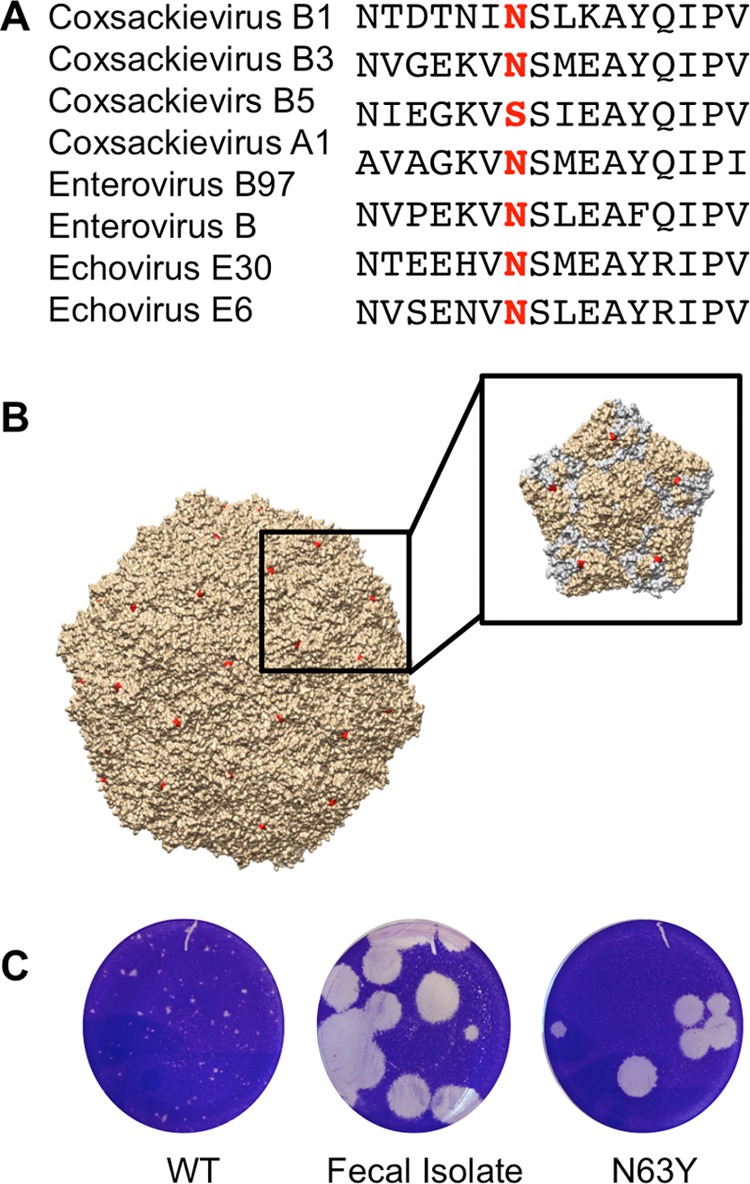
VP3 N63Y is sufficient for the large-plaque phenotype. RT-PCR products from several large plaques were sequenced and found to contain the VP3 N63Y mutation. The N63Y mutant was cloned into a new infectious clone, and virus was generated. (A) Alignment of the VP3-63 region sequences of various viruses. The highlighted residue is in position 63. (B) CVB3 structure with the location of VP3-63 in red. The inset shows one 5-fold symmetry axis, with the VP3 proteins in gray. (C) Plaque phenotypes of WT CVB3, a plaque-purified fecal isolate with the large-plaque phenotype, and N63Y mutant CVB3.

To identify the mutation(s) responsible for the large-plaque phenotype, we isolated six individual large-plaque viruses from mouse feces, performed plaque purification to generate large-plaque “fecal isolate” samples, extracted RNA, performed reverse transcription (RT)-PCR, and sequenced products by consensus sequencing. We sequenced the capsid-coding region with the rationale that major changes in plaque size frequently map to capsid proteins (35, 36). Sequence alignment revealed several silent point mutations and two amino acid changes in the capsid-coding region of large plaques: T151S in VP2 and N63Y in VP3. Sequence alignment revealed that amino acid 151 of VP2 is a variable position, with T or S present in many GenBank samples. However, N63 is highly conserved among coxsackieviruses and echoviruses (Fig. 3A) and is located on the virion surface near the DAF binding site (Fig. 3B) (37). Using reverse genetics, we cloned N63Y into the CVB3 infectious clone and found that N63Y is sufficient for the large-plaque phenotype (Fig. 3C). Recently, Bordería et al. identified polymorphisms at position 63 of VP3, including N63Y, N63D, and N63H, that emerged following viral passage in A549 human lung cells (38). Therefore, VP3-63 may affect viral replication in different cell types. Because N63Y was sufficient for the large-plaque phenotype, we examined the properties of N63Y-containing viruses.

### N63Y mutant CVB3 has a growth defect in cell culture.

**FIG 4:**
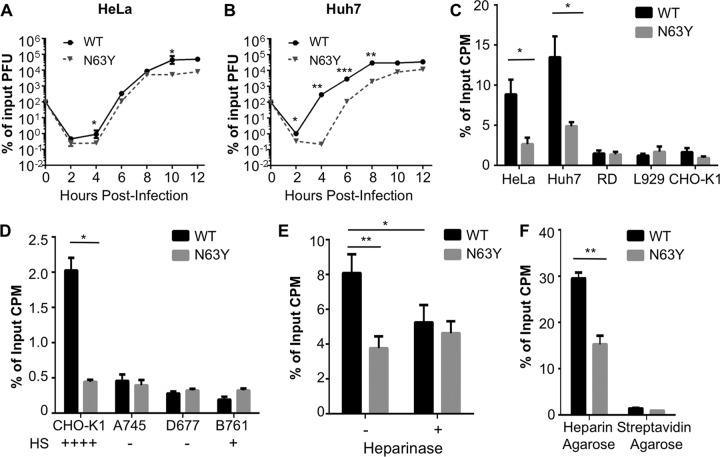
N63Y mutant CVB3 has a growth defect in cell culture and reduced glycan-mediated cell attachment. Single-cycle assays of viral replication in HeLa (A) and Huh7 (B) cells were performed. Infections with WT or N63Y mutant CVB3 were performed at an MOI of 0.1. Viral titers were determined by plaque assay with HeLa cells. *n* = 3. (C) Cell attachment of ^35^S-labeled WT or N63Y mutant CVB3. Virus was incubated with cells at 4°C for 40 min. Cells were washed and trypsinized, and cell-associated ^35^S was quantified. (D) ^35^S-labeled WT or N63Y mutant CVB3 was incubated with CHO cells (CHO-K1, pgsA745, pgsD677, and pgsB761) that vary in GAG expression. Plus and minus signs indicate the relative levels of GAGs on the cell surface. (E) Effect of heparinase treatment on CVB3 cell attachment. Huh7 cells were treated with or without heparinase I for 90 min prior to quantification of ^35^S CVB3 attachment. *n* = 7. (F) Heparin-agarose pulldown assay. ^35^S-labeled WT or N63Y mutant CVB3 was incubated with heparin-agarose resin or streptavidin-agarose resin (control). Resin was washed, and bound ^35^S-labeled CVB3 was quantified. *n* = 3. *, *P* < 0.05; **, *P* < 0.01; ***, *P* < 0.001.

To characterize N63Y mutant CVB3, we first compared the growth of N63Y and WT CVB3 in cell culture by using single-cycle growth curve assays. Three human cell lines, HeLa, Huh7, and RD, and two rodent cell lines, CHO-K1 and L929, were infected at a multiplicity of infection (MOI) of 0.1, and viral titers were determined over time. N63Y mutant CVB3 demonstrated a growth defect in most of the cell lines (Fig. 4A and B; see Fig. S1 in the supplemental material). Furthermore, both WT and N63Y mutant CVB3 replicated better in human cell lines than in rodent cell lines. These cell lines have different levels of CVB3 receptor expression (39–41). Therefore, we hypothesized that the replication differences between WT and N63Y mutant CVB3 may be due to altered cell attachment and receptor binding.

### N63Y mutant CVB3 binds cells and GAGs less efficiently than WT CVB3 does.

To determine why N63Y mutant CVB3 has a growth defect in cell culture, we first examined cell attachment. For the cell attachment assay, ^35^S-labeled CVB3 was incubated with HeLa, Huh7, RD, L929, and CHO-K1 cell layers at 4°C to facilitate binding but not internalization. After washing, we quantified the cell-associated ^35^S label. N63Y mutant CVB3 showed significantly reduced attachment to HeLa and Huh7 cells (Fig. 4C), and both N63Y and WT CVB3 showed limited attachment to RD, L929, and CHO-K1 cells. Therefore, the efficiency of viral attachment correlates with growth in cell culture.

We next examined whether binding to CAR or DAF differs between WT and N63Y mutant CVB3. ^35^S-labeled WT or N63Y mutant CVB3 was incubated with nitrocellulose membranes containing immobilized human or murine CAR (hCAR or mCAR, respectively) or human or murine DAF (hDAF or mDAF, respectively). After washing, the amount of virus bound was quantified by phosphorimager analysis (42). We found that WT and N63Y mutant CVB3 bound hCAR and mCAR equivalently (see Fig. S2 in the supplemental material) and neither virus bound hDAF or mDAF (data not shown).

Because altered binding to known CVB3 proteinaceous receptors could not explain the attachment defect of N63Y mutant CVB3, we quantified viral binding to GAGs. First, we performed the cell attachment assay with three cell lines derived from CHO-K1 cells that have different levels of HS on their surface because of knockout of host proteins involved in GAG synthesis (39, 40): GAG-negative pgsA745, HS-negative pgsD677, and 5% HS pgsB761. While N63Y mutant CVB3 showed significantly less attachment to CHO-K1 cells than WT CVB3 did, it showed attachment comparable to that of WT CVB3 in the other three cell lines (Fig. 4D). These data indicate that N63Y mutant CVB3 may have altered binding to GAGs. Second, we treated Huh7 cells with heparinase to remove GAGs and examined viral attachment. Heparinase treatment reduced the attachment of WT CVB3 to cells, with little effect on N63Y mutant CVB3 attachment (Fig. 4E). Third, we quantified CVB3 binding to GAGs by a direct method, heparin-agarose pulldown assays. ^35^S-labeled CVB3 was incubated with heparin-agarose resin or control streptavidin-agarose resin at 37°C for 3 h. After washing, resin-associated ^35^S was quantified. We found that N63Y mutant CVB3 bound less efficiently to heparin-agarose than WT CVB3 did (Fig. 4F).

On the basis of the reduced binding of N63Y mutant CVB3 to heparin-agarose and previous studies showing that sulfated glycans in agar can inhibit plaque formation by some viruses (43), we hypothesized that WT CVB3 binds sulfated glycans in agar overlays, which reduces plaque size. Conversely, we hypothesized that N63Y mutant CVB3 plaque formation would not be inhibited by sulfated glycans in agar. To test this, we examined the plaque sizes of WT and N63Y mutant CVB3 by using agar versus agarose overlays in the presence of added HS or heparin. Agarose contains low levels of sulfated glycans, and WT and N63Y mutant CVB3 generated similar-size large plaques under an agarose overlay. Addition of HS or heparin to the overlay reduced the WT plaque size but not the N63Y mutant plaque size (see Fig. S3 in the supplemental material). Taken together, these data suggest that the N63Y mutant has reduced binding to sulfated glycans, which facilitates the formation of large plaques in the presence of sulfated glycans but reduces its attachment to cell surface GAGs and growth in cell culture.

### N63Y mutant CVB3 has increased replication and pathogenesis in mice.

**FIG 5:**
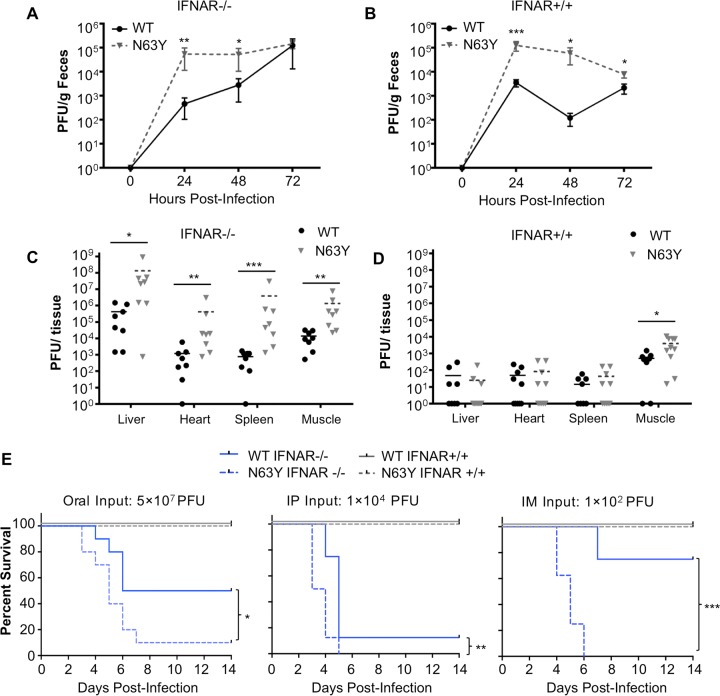
N63Y mutant CVB3 shows enhanced replication and virulence in mice. The viral fecal shedding profiles of IFNAR^−/−^ (A) or IFNAR^+/+^ (B) mice orally inoculated with 5 × 10^7^ PFU of WT or N63Y mutant CVB3 were determined. Virus titers were determined by plaque assay. Tissue viral titers at 72 hpi were determined in IFNAR^−/−^ (C) or IFNAR^+/+^ (D) mice orally inoculated with 5 × 10^7^ PFU of WT or N63Y mutant CVB3. (E) Survival curves of CVB3-infected mice. From left to right, IFNAR^−/−^ (blue lines) or IFNAR^+/+^ (gray lines) mice were inoculated orally with 5 × 10^7^ PFU, i.p. inoculated with 1 × 10^4^ PFU, or i.m. inoculated with 1 × 10^2^ PFU of WT (solid line) or N63Y mutant (dashed line) CVB3. For all panels, *n* = 5 to 8. *, *P* < 0.05; **, *P* < 0.01; ***, *P* < 0.001.

Given that N63Y mutant CVB3 emerged following replication in mice, we hypothesized that it may have a fitness advantage *in vivo*. To test this hypothesis, we first examined viral replication in IFNAR^−/−^ mice orally inoculated with 5 × 10^7^ PFU of WT or N63Y mutant CVB3. Fecal samples were collected at 24, 48, and 72 hpi, and titers were determined by plaque assay. N63Y mutant CVB3 had higher viral titers in feces than WT CVB3 did, especially at early time points (Fig. 5A). Since it was shown previously that WT CVB3 has limited replication in immunocompetent mice, we wondered whether N63Y mutant CVB3 would have enhanced replication in IFNAR^+/+^ mice. To test this, we orally inoculated IFNAR^+/+^ mice and observed that the N63Y mutant replicated more efficiently than WT CVB3 (Fig. 5B). We also examined viral titers in various tissues from orally inoculated IFNAR^+/+^ or IFNAR^−/−^ mice at 72 hpi. N63Y mutant CVB3 titers were significantly higher than WT CVB3 titers in all of the IFNAR^−/−^ mouse tissues examined (Fig. 5C). While viral titers were low in most IFNAR^+/+^ mouse tissues, N63Y mutant CVB3 titers were significantly higher than WT CVB3 titers in leg muscle, indicating that the N63Y mutant was more capable of dissemination to body sites distant from the gut in immunocompetent animals (Fig. 5D). These results indicate that N63Y mutant CVB3 has enhanced replication and systemic dissemination in orally inoculated mice, including immunocompetent mice.

To examine the virulence of WT versus N63Y mutant CVB3, we monitored the survival of mice following infection via different inoculation routes. We infected IFNAR^−/−^ and IFNAR^+/+^ mice orally with 5 × 10^7^ PFU, i.p. with 1 × 10^4^ PFU, or i.m. with 1 × 10^2^ PFU of CVB3 (Fig. 5E). First, we found that IFNAR^+/+^ mice did not succumb to CVB3 infection regardless of the virus or inoculation route. Second, we found that IFNAR^−/−^ mice were most susceptible to i.m. infection (100 PFU) and least susceptible to oral infection (5 × 10^7^ PFU). Third, we found that N63Y mutant CVB3-infected mice had a lower survival rate than WT CVB3-infected mice. In summary, N63Y mutant CVB3 replicated and disseminated more efficiently in both IFNAR^+/+^ and IFNAR^−/−^ mice and was more pathogenic than WT CVB3 in IFNAR^−/−^ mice.

### N63Y mutant CVB3 has enhanced dissemination to the liver and induces more liver damage than WT CVB3.

**FIG 6:**
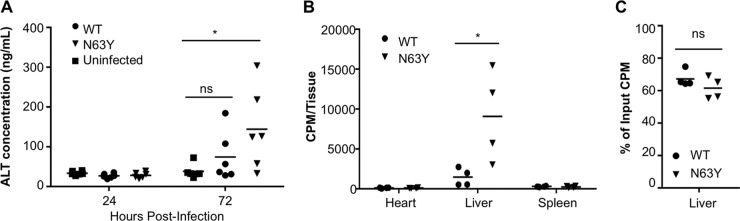
N63Y mutant CVB3 shows greater dissemination to the liver and induces more liver damage than WT CVB3. IFNAR^−/−^ mice were orally infected with 5 × 10^7^ PFU of WT or N63Y mutant CVB3. (A) Blood was harvested at 24 and 72 hpi, and ALT levels were quantified by ELISA. *n* = 6. (B) IFNAR^−/−^ mice were inoculated i.p. with 2 × 10^7^ PFU/20,000 cpm ^35^S labeled WT or N63Y mutant CVB3. Heart, liver, and spleen samples were harvested at 1 hpi, and tissue-associated counts per minute were quantified. (C) Binding of CVB3 to homogenized tissues. A total of 3 × 10^6^ PFU/3,000 cpm ^35^S labeled WT or N63Y mutant CVB3 were incubated with homogenized liver tissue from IFNAR^−/−^ mice for 60 min; this was followed by washing and quantification of the tissue-associated counts per minute. *, *P* < 0.05; **, *P* < 0.01; ***, *P* < 0.001; ns, not significant.

To determine the cause of death of CVB3-infected IFNAR^−/−^ mice and understand why N63Y mutant CVB3 is more pathogenic than WT CVB3, we examined liver pathology. Wessely et al. demonstrated that CVB3-infected IFNAR^−/−^ mice develop liver pathology (44). We orally inoculated IFNAR^−/−^ mice with 5 × 10^7^ PFU of WT or N63Y mutant CVB3. We collected blood at 24 and 72 hpi to measure serum protein levels and harvested livers at 72 hpi for histological analysis. First, we measured the levels of alanine aminotransferase (ALT), a marker of liver damage, in serum. ALT levels were low at 24 hpi in all of the mice (Fig. 6A). However, ALT levels were significantly elevated at 72 hpi in mice infected with N63Y mutant CVB3 compared with uninfected controls. While 33% of the mice infected with WT CVB3 had higher ALT levels than uninfected mice, 66% of the mice infected with N63Y mutant CVB3 had higher ALT levels than uninfected mice. Second, we performed histological analysis of liver tissue harvested from mice at 72 hpi. As shown in Fig. S4 in the supplemental material, mice infected with N63Y mutant CVB3 had more severe liver pathology than uninfected or WT CVB3-infected mice. Histological examination revealed hepatic cell necrosis with extensive polymorphonuclear cell infiltration at 72 hpi in N63Y mutant CVB3-infected mice. Moderate polymorphonuclear cell infiltration was observed in WT CVB3-infected mice. In agreement with Wessely et al., our data suggest that CVB3-infected IFNAR^−/−^ mice develop liver damage (44). Furthermore, our data suggest that mice infected with N63Y mutant CVB3 have more liver damage than WT CVB3-infected animals.

Because of the enhanced liver damage observed in N63Y mutant CVB3-infected mice, we hypothesized that N63Y mutant CVB3 may have increased homing to the liver. To test this, we i.p. injected IFNAR^−/−^ mice with ^35^S-labeled WT or N63Y mutant CVB3, collected their hearts, livers, and spleens at 1 hpi, and quantified the amounts of tissue-associated virus by scintillation counting. While WT and N63Y mutant CVB3 were present at equivalent levels in the heart and spleen, there was 8-fold more N63Y mutant CVB3 present in the liver (Fig. 6B). In fact, on average, half of the injected N63Y mutant CVB3 was found in the liver. These effects were observed in livers harvested at 1 hpi, hours before a single cycle of viral replication can occur. The elevated amount of N63Y mutant CVB3 found in the liver could be due to increased binding to liver tissue and/or increased dissemination to the liver. To distinguish between these possibilities, we quantified the binding of ^35^S-labeled WT versus N63Y mutant CVB3 to liver tissue homogenates from IFNAR^−/−^ mice *ex vivo*. We found equivalent binding of WT and N63Y mutant CVB3 to liver tissue homogenates (Fig. 6C). Therefore, these data suggest that N63Y mutant virions disseminate to the liver more efficiently than WT virions do.

## DISCUSSION

Because of error-prone RNA replication, RNA viruses generate mutations in every single cycle of propagation and selective pressure dictates which variants are present in the population. In this study, we discovered the emergence of a CVB3 mutant *in vivo*, examined its properties *in vitro* and *in vivo*, and used it as a platform to understand viral adaptation and factors that influence the emergence of variants.

Many viruses attach to cells by binding HS or other GAGs (45). For some viruses, GAGs are *bona fide* receptors. Other viruses have nonglycan proteinaceous receptors, but binding to GAGs can enhance attachment. GAGs most commonly interact with viral particles via positively charged amino acids such as lysine or arginine (27, 28, 41, 46). Therefore, the N63Y amino acid change observed here is slightly unusual. It is possible that a change from asparagine to tyrosine disrupts GAG binding to a nearby site on the viral capsid or that noncanonical GAG-capsid interactions occur.

Acquisition of HS binding is a common cell culture adaptation for several viruses. In several cases, cell culture adaptation to bind HS attenuates viruses *in vivo*. For example, cell culture adaptation of foot-and-mouth disease virus selects viruses that bind HS, but these viruses are attenuated in cattle (29). A cell culture-adapted vaccine strain of chikungunya virus is attenuated because of increased GAG binding (37). A dengue virus variant with reduced HS binding caused severe disease in mice, with reduced clearance from serum (47). Similarly, laboratory Sindbis virus strains bind HS (28); however, HS-binding Sindbis virus strains are attenuated in mice. Interestingly, Byrnes et al. isolated a large-plaque variant of Sindbis virus from mice inoculated with an HS-binding strain. The large-plaque variant bound HS less efficiently and was more virulent in mice (48). The non-HS-binding variant had increased viremia and decreased clearance from serum, perhaps because of reduced clearance by heparin-binding proteins in the host. These Sindbis virus results are very reminiscent of our CVB3 results.

Our results demonstrate that WT CVB3 plaque formation is inhibited by substances, likely sulfated glycans, in agar, whereas N63Y mutant CVB3 is not. Importantly, if we had used agarose rather than agar for plaque assay overlays, we would not have observed the emergence of N63Y mutant CVB3 in mice. Perhaps this explains why other groups have not yet reported the emergence of N63Y variants in animal studies. We only identified the emergence of this mutant because of a very obvious phenotypic change in plaque size. It is possible, and even likely, that the emergence of variants occurs frequently in animals but may be missed because of a lack of observable phenotypes.

The emergence of the large-plaque variant varied when we used different inoculation routes. We saw large-plaque variants emerge in fecal viruses from orally inoculated mice and tissues from i.p. injected mice but in not muscle tissue from i.m. injected IFNAR^−/−^ mice. However, we observed large-plaque viruses in liver from i.m. injected IFNAR^−/−^ mice. Additionally, more large-plaque variants emerged from IFNAR^+/+^ mice than from IFNAR^−/−^ mice. These results suggest that increased selective pressure in the intestine and peritoneal cavity may have facilitated the emergence of large-plaque variants, particularly in immunocompetent mice. Alternatively, it is possible that N63Y mutant CVB3 has a fitness advantage only in certain tissues.

N63Y mutant CVB3 had enhanced replication and pathogenesis in mice regardless of the inoculation route. We do not know whether the GAG-binding deficiency of N63Y mutant CVB3 is sufficient for its enhanced replication and pathogenesis or if other effects contribute. In agreement with a previous study, we found that IFNAR^−/−^ mice developed liver pathology upon CVB3 infection. Liver pathology was more severe in mice infected with N63Y mutant CVB3, potentially because N63Y mutant CVB3 had increased dissemination to the liver (Fig. 6). Whether the increased viral shedding in mice inoculated orally with N63Y mutant CVB3 is due to altered viral dissemination to the liver or other tissues is unknown. It is possible that the enhanced replication and pathogenesis of N63Y mutant CVB3 are specific to mice, since this variant is not observed in human clinical isolates. However, because N63Y mutant CVB3 is more pathogenic than WT CVB3 in orally inoculated immunodeficient mice and N63Y mutant CVB3 replicates well in orally inoculated immunocompetent mice and even disseminates to muscle tissue, it may be useful as a new model system for examining CVB3 replication and pathogenesis by using the natural route of infection.

## MATERIALS AND METHODS

### Cells and virus.

HeLa cells were propagated in Dulbecco’s modified Eagle’s medium (DMEM) supplemented with 10% calf serum. CHO-K1 cells and mutant CHO cells (pgsA745, pgsB761, and pgsD677) were obtained from Jeffrey Esko (University of California, San Diego) and maintained in F12/DMEM supplemented with 5% fetal bovine serum. Human rhabdomyosarcoma (RD) cells, Huh7 cells, 293 cells, and murine L929 cells were maintained in DMEM supplemented with 5% fetal bovine serum.

The CVB3 Nancy infectious clone was obtained from Marco Vignuzzi (Pasteur Institute, Paris, France), who received it from Reinhard Kandolf. This version of CVB3 Nancy binds HS (36). Stocks of CVB3 were prepared in HeLa cells by cotransfection of the infectious clone plasmid and a plasmid expressing T7 RNA polymerase (49). Virus titers were determined by plaque assay with HeLa cells as previously described (49). Agar (1% final concentration; Becton Dickinson) was used in plaque assay overlays unless otherwise indicated. Plaque size was quantified with ImageJ 1.47v software. To determine whether fecal viruses were input/inoculum virus or virus that had undergone replication, we used neutral-red-labeled, light-sensitive CVB3 as described previously (50). To determine the percentage of replicated virus, samples were processed in the dark and a portion was light exposed. The percentage of replicated virus was calculated by dividing the light-exposed number of PFU/ml by the non-light-exposed number of PFU/ml and multiplying the result by 100. ^35^S-labeled CVB3 was prepared as previously described (30). The specific activity of ^35^S-labeled CVB3 stocks was approximately 1,000 PFU/cpm.

### Sequencing of fecal isolates, cloning of N63Y mutant CVB3, and structure/sequence alignments.

Six individual plaques generated from fecal samples were plaque purified to generate independent “fecal isolates.” Viral RNA was isolated and purified with the RNeasy minikit (Qiagen). RT-PCR was performed with SuperScript II reverse transcriptase (Invitrogen) by using primer 5′ CGCCACGGACGAGGTA 3′ to generate cDNA. PCRs were performed with primer pairs that cover most of the VP1-VP3 coding region, i.e., sense primer 5′ GCTGCCCGATGCTTTGT 3′, antisense primer 5′ GGCCGAACCACAGAACATAAAC 3′, sense primer 5′ GCAATATGACGTCACACCAGAGAT 3′, antisense primer 5′ AGGGCTGTTGAGTACTTGTTATGA 3′, sense primer 5′ ACTACCGGTTTGTTGCTTCAGATC 3′, and antisense primer 5′ CTACTAGACCTGGTCCTTCAAACGA 3′. PCR products were sequenced by the University of Texas (UT) Southwestern Sequencing Core. All six isolates contained two amino acid changes, VP3 N63Y (an A-to-T mutation at position 1956) and VP2 T151S (a C-to-G mutation at position 1432 and a G-to-C mutation at position 1433), as well as other silent point mutations. Because position 151 of VP2 is highly variable in CVB3 sequences, we focused our efforts on VP3 N63Y. A 377-bp fragment containing the N63Y mutation was generated by BsiWI/BglII digestion of an RT-PCR product from one fecal isolate, and this fragment was cloned into a new CVB3 Nancy plasmid from nucleotide 1695 (BsiWI) to nucleotide 2072 (BglII). The new plasmid was then sequenced, and N63Y was confirmed as the only mutation in the PCR-generated region. The large-plaque phenotype of this new clone was confirmed by plaque assay. The structure in Fig. 3 was modified from a previously determined crystal structure (Protein Data Bank code 1cov) (51). The sequences of the region near residue 63 of VP3 from different viruses were aligned by the PROMALS3D multiple-sequence and -structure alignment server (52).

### Single-cycle growth curve assay.

Six-centimeter plates were each seeded with 1 × 10^6^ HeLa, Huh7, RD, CHO, or L929 cells. Each plate was inoculated with WT or N63Y mutant CVB3 at an MOI of 0.1. After 30 min of incubation at 37°C, the inoculum was aspirated from the cells. The cell monolayers were washed, and 3 ml of fresh medium was added. At 2, 4, 6, 8, 10, and 12 h after infection, cells were trypsinized and pelleted. To release intracellular virus, cell pellets were freeze-thawed three times before the virus titer was determined by plaque assay with HeLa cells. All data were compared to input stock titers, which were set to 100%.

### CVB3 cell attachment assay.

^35^S-labeled CVB3 (3 × 10^6^ PFU/3,000 cpm) was incubated with 8.8 × 10^6^ HeLa, Huh7, RD, CHO, or L929 cells at 4°C for 40 min to facilitate viral binding. Cells were washed three times with cold phosphate-buffered saline (PBS) and trypsinized, and ^35^S was quantified in a scintillation counter. For certain experiments, cells were treated with 10 U/ml heparinase I (Sigma Aldrich) in 10 mM phosphate buffer (pH 7.4) containing 0.15 M NaCl, 3 mM KCl, 0.5 mM MgCl_2_, 1 mM CaCl_2_, 0.1% glucose, 1% fetal calf serum, and 0.5% bovine serum albumin for 90 min prior to the viral attachment assay (53).

### Heparin, CAR, and DAF binding assays.

^35^S-labeled CVB3 (5 × 10^6^ PFU/5,000 cpm) was mixed with heparin agarose resin (Sigma Aldrich) or streptavidin agarose resin (Sigma Aldrich). Samples were incubated for 3 h at 37°C. Following incubation, resin was pelleted by centrifugation at 2,600 × *g* for 2 min. Resin was washed three times with PBS+ (PBS supplemented with 100 μg/ml CaCl_2_ and 100 μg/ml MgCl_2_), and resin-associated radioactivity was quantified by scintillation counting. Data are expressed as a percentage of the input counts per minute ([resin-associated counts per minute/input counts per minute] × 100). For CAR and DAF binding assays, human (R&D Systems) or murine CAR (U.S. Biological) or human (R&D Systems) or murine DAF (Sino Biological) was immobilized on nitrocellulose membranes and the membranes were incubated with ^35^S-labeled CVB3 (6 × 10^7^ PFU/60,000 cpm) at 37°C for 12 h (38). After the membranes were washed, membrane-associated radioactivity was quantified by phosphorimager analysis.

### Mouse experiments.

All animals were handled according to the Guide for the Care of Laboratory Animals of the National Institutes of Health, All mouse studies were performed at UT Southwestern (Animal Welfare Assurance no. a3472-01) by using protocols approved by the local Institutional Animal Care and Use Committee in a manner designed to minimize pain, and any animals that exhibited severe disease were euthanized immediately with isoflurane. C57BL/6 PVR IFNAR^+/+^ and C57BL/6 PVR IFNAR^−/−^ mice were obtained from S. Koike (Tokyo, Japan) (54), and all experimental mice were 4- to 10-week-old males. In the experiments shown in Fig. 1, mice were orally inoculated with 5 × 10^7^ PFU of neutral-red-labeled, light-sensitive CVB3 and fecal samples were collected in the dark. In the experiments shown in Fig. 2, mice were orally, i.p. inoculated with 5 × 10^7^ PFU of WT CVB3 or i.m. inoculated with 2 × 10^6^ PFU of WT CVB3, tissues (muscle, liver, heart, and/or spleen) were harvested at 48 hpi, and fecal samples were collected from orally inoculated mice at 72 hpi. In the experiments shown in Fig. 5, mice were orally inoculated with 5 × 10^7^ PFU of CVB3 or inoculated i.p. or i.m. with 1 × 10^4^ PFU and 1 × 10^2^ PFU of CVB3, respectively. Tissue samples were processed as previously described (30). In the experiments shown in Fig. 6A, mice were orally inoculated with 5 × 10^7^ PFU of CVB3 and blood was collected at 24 hpi by cheek bleed and at 72 hpi by heart puncture. Following heart puncture, mice were perfused with 4% paraformaldehyde, liver tissue was collected and fixed in 4% paraformaldehyde for 48 h, and hematoxylin-and-eosin (H&E) staining was performed by the UT Southwestern Molecular Pathology Core. For ALT quantification, serum was separated by centrifugation and ALT concentrations were determined by enzyme-linked immunosorbent assay (ELISA; MyBiosource) according to the manufacturer’s instructions. In the experiments shown in Fig. 6B, mice were inoculated i.p. with ^35^S-labeled WT or N63Y mutant CVB3 at 2 × 10^7^ PFU/20,000 cpm and tissues were collected 1 hpi for quantification in a scintillation counter. In all cases, feces and tissue samples were processed prior to viral titer assay or scintillation counting as previously described (30). In the experiments shown in Fig. 6C, fresh tissues were homogenized with a Bullet Blender homogenizer (Next Advanced Inc., Averill Park, NY) (55) prior to incubation with 3 × 10^6^ PFU/3,000 cpm of ^35^S-labeled WT or N63Y mutant CVB3. After a 1-h incubation at 37°C, tissues were washed with PBS+ and tissue-associated radioactivity was quantified by scintillation counting.

### Statistical analysis.

Data throughout are shown as the mean ± the standard error of the mean. The differences between groups were examined by unpaired two-tailed Student *t* tests. *P* < 0.05 was considered statistically significant. All analyses were performed with Graph Pad Prism 6.03 software (GraphPad Software, La Jolla, CA).

### Ethics/biosafety statement.

During the course of this study, we saw a large-plaque CVB3 variant emerge in mice and unexpectedly found that this mutant virus had increased replication and virulence in mice. Upon observing the enhanced virulence of this virus, we consulted with the UT Southwestern Biological and Chemical Safety Office. It was determined that our existing Animal Biosafety Level 2+ protocols, the same used for our poliovirus work, were appropriate for these experiments.

## SUPPLEMENTAL MATERIAL

Figure S1  N63Y mutant CVB3 has a growth defect in several cell lines. Single-cycle assays of viral replication in RD (A), CHO (B), and L929 (C) cells. Infections with WT or N63Y mutant CVB3 were performed at an MOI of 0.1. Viral titers were determined by plaque assay with HeLa cells. Download Figure S1, TIF file, 0.6 MB

Figure S2  N63Y mutant CVB3 does not show altered binding to human or murine CAR. ^35^S-labeled WT or N63Y mutant CVB3 was incubated with nitrocellulose membranes containing immobilized hCAR or mCAR protein. After washing, the bound viral counts per minute were determined to quantify binding. *n* = 3. Download Figure S2, TIF file, 2.4 MB

Figure S3  Sulfated glycans/GAGs inhibit WT CVB3 plaque formation. Plaque assays were performed with WT or N63Y mutant CVB3 by using agar and agarose overlays in the presence or absence of 0.1 mg/ml HS or heparin. Plaque sizes were quantified with ImageJ. For ease of comparison, data are displayed as fold WT plaque size, which is the size of each plaque divided by the average size of all WT plaques from plates with agar overlays (average = 0.2473 mm^2^). Download Figure S3, TIF file, 0.7 MB

Figure S4  Histopathology of liver tissue. IFNAR^−/−^ mice were orally infected with 5 × 10^7^ PFU of WT or N63Y mutant CVB3. At 72 hpi, liver tissue was collected and H&E staining was performed. Tissue samples from two representative mice per condition are shown. Download Figure S4, TIF file, 2.6 MB
