# Supplementary figures and images for: Emergence of a Large-Plaque Variant in Mice Infected with Coxsackievirus B3
Source: mBio. 2016 Mar 29;7(2):e00119-16. doi: 10.1128/mBio.00119-16 (PMC4817249; doi:10.1128/mBio.00119-16)

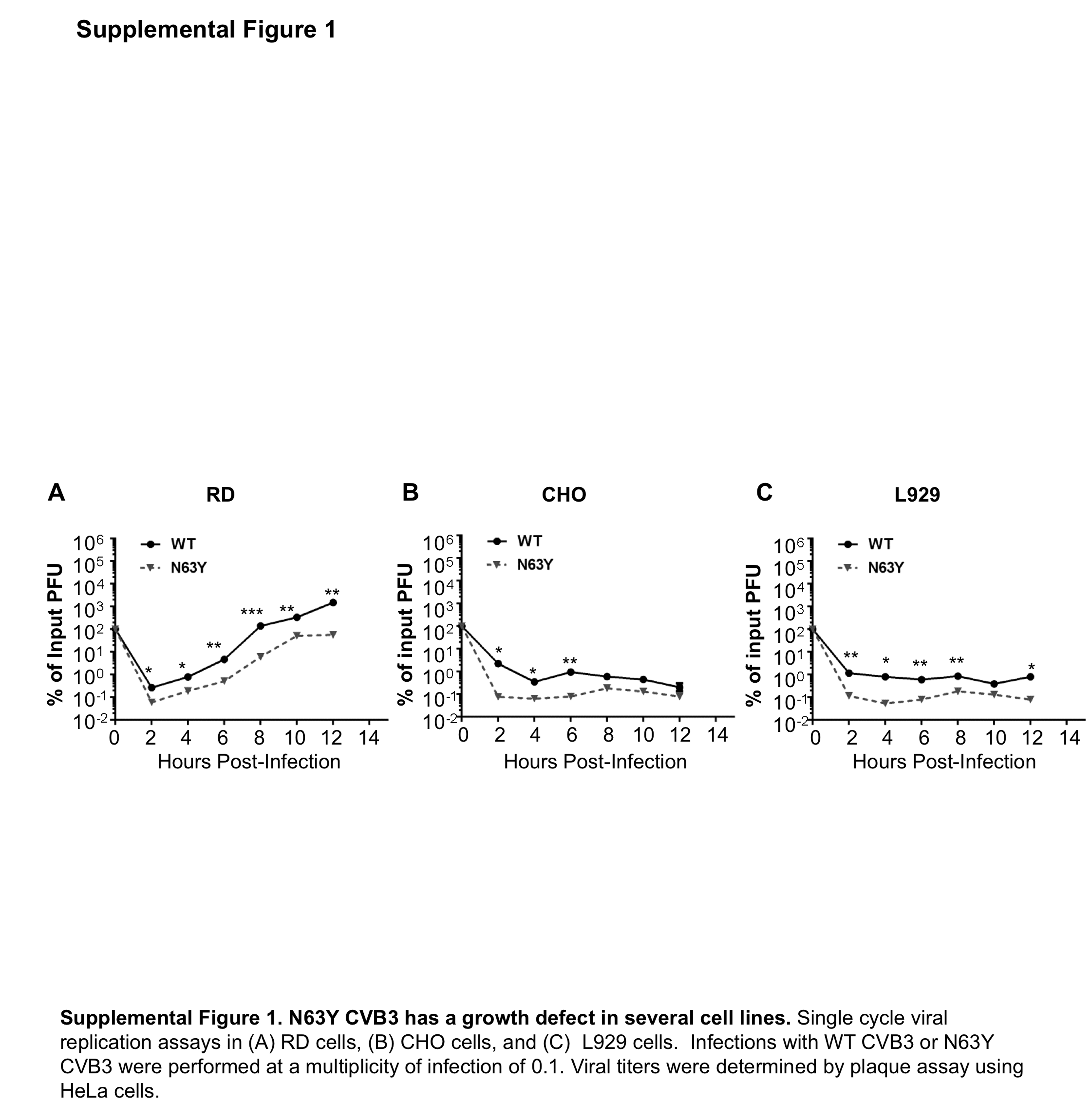

Supplement: Figure S1 — N63Y mutant CVB3 has a growth defect in several cell lines. Single-cycle assays of viral replication in RD (A), CHO (B), and L929 (C) cells. Infections with WT or N63Y mutant CVB3 were performed at an MOI of 0.1. Viral titers were determined by plaque assay with HeLa cells. Download [file mbo002162746sf1.tif]

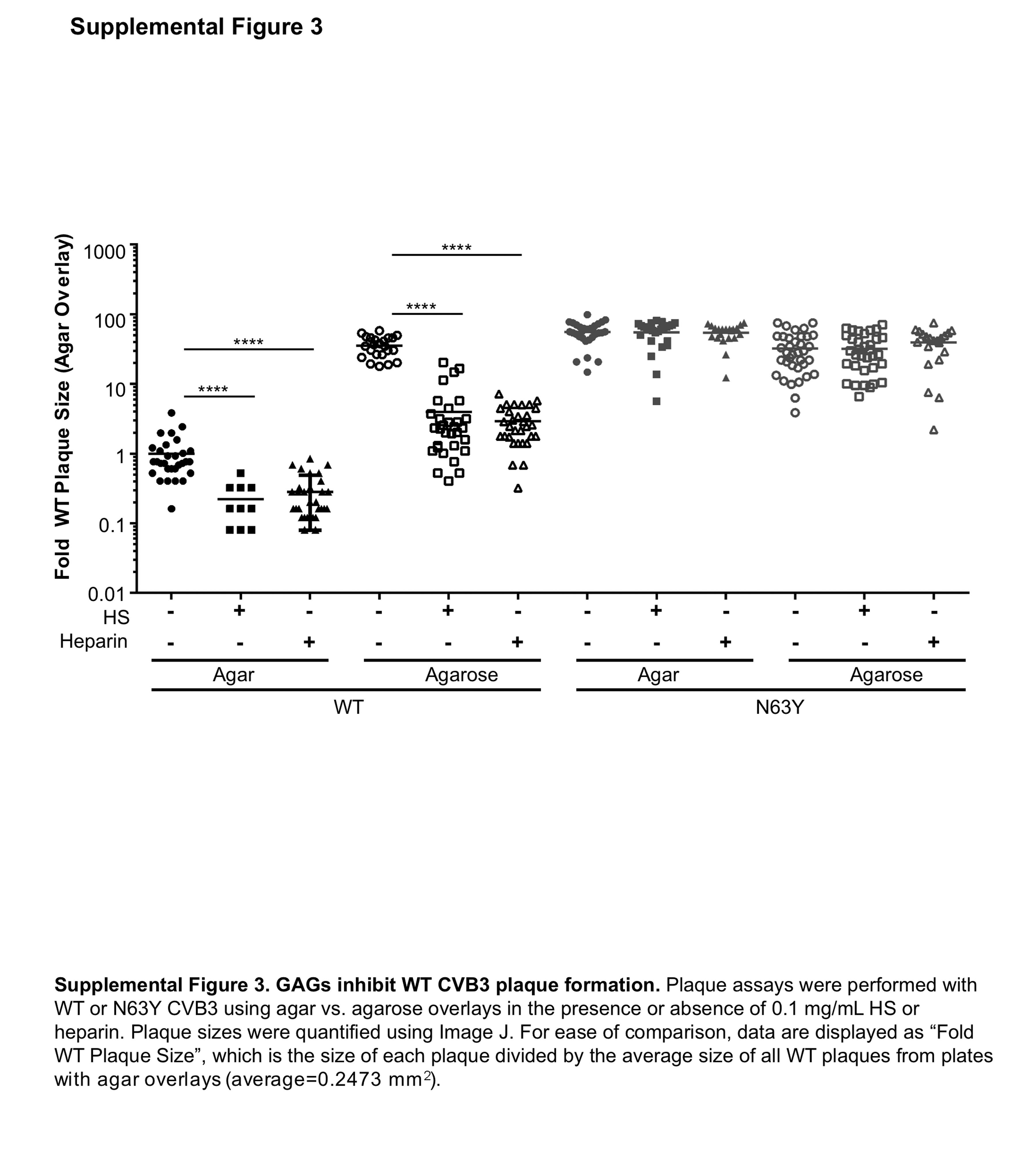

Supplement: Figure S3 — Sulfated glycans/GAGs inhibit WT CVB3 plaque formation. Plaque assays were performed with WT or N63Y mutant CVB3 by using agar and agarose overlays in the presence or absence of 0.1 mg/ml HS or heparin. Plaque sizes were quantified with ImageJ. For ease of comparison, data are displayed as fold WT plaque size, which is the size of each plaque divided by the average size of all WT plaques from plates with agar overlays (average = 0.2473 mm2). Download [file mbo002162746sf3.tif]

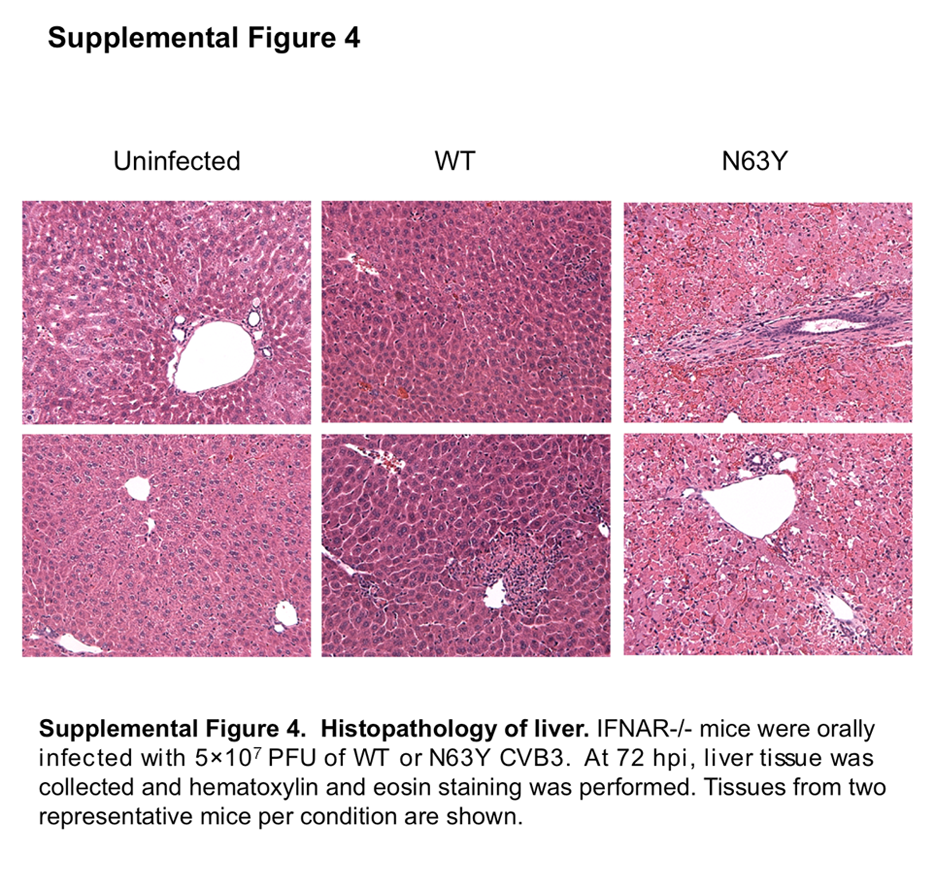

Supplement: Figure S4 — Histopathology of liver tissue. IFNAR−/− mice were orally infected with 5 × 10^7^ PFU of WT or N63Y mutant CVB3. At 72 hpi, liver tissue was collected and H&E staining was performed. Tissue samples from two representative mice per condition are shown. Download [file mbo002162746sf4.tif]
